# Lipid Environment Modulates the Development of Acute Tolerance to Ethanol in *Caenorhabditis elegans*

**DOI:** 10.1371/journal.pone.0035192

**Published:** 2012-05-04

**Authors:** Jill C. Bettinger, Kapo Leung, Mia H. Bolling, Andrew D. Goldsmith, Andrew G. Davies

**Affiliations:** 1 Department of Pharmacology and Toxicology, Virginia Commonwealth University, Richmond, Virginia, United States of America; 2 Department of Psychiatry, Virginia Commonwealth University, Richmond, Virginia, United States of America; 3 Virginia Commonwealth University - Alcohol Research Center, Virginia Commonwealth University, Richmond, Virginia, United States of America; 4 Ernest Gallo Clinic and Research Center, University of California San Francisco, Emeryville, California, United States of America; Brown University, United States of America

## Abstract

The development of tolerance to a drug at the level of the neuron reflects a homeostatic mechanism by which neurons respond to perturbations of their function by external stimuli. Acute functional tolerance (AFT) to ethanol is a fast compensatory response that develops within a single drug session and normalizes neuronal function despite the continued presence of the drug. We performed a genetic screen to identify genes required for the development of acute functional tolerance to ethanol in the nematode *C. elegans*. We identified mutations affecting multiple genes in a genetic pathway known to regulate levels of triacylglycerols (TAGs) via the lipase LIPS-7, indicating that there is an important role for TAGs in the development of tolerance. Genetic manipulation of *lips-7* expression, up or down, produced opposing effects on ethanol sensitivity and on the rate of development of AFT. Further, decreasing cholesterol levels through environmental manipulation mirrored the effects of decreased TAG levels. Finally, we found that genetic alterations in the levels of the TAG lipase LIPS-7 can modify the phenotype of gain-of-function mutations in the ethanol-inducible ion channel SLO-1, the voltage- and calcium-sensitive BK channel. This study demonstrates that the lipid milieu modulates neuronal responses to ethanol that include initial sensitivity and the development of acute tolerance. These results lend new insight into studies of alcohol dependence, and suggest a model in which TAG levels are important for the development of AFT through alterations of the action of ethanol on membrane proteins.

## Introduction

Alcohol abuse is a prevalent and serious disorder. Current drug treatments are inadequate, in part because the molecular nature of acute ethanol response is not well understood. A component of the ethanol response in humans that is predictive of the susceptibility to abuse alcohol is an individual’s naïve level of response to the drug [1], and this phenotype is strongly genetically influenced [2], [3]. This level of response to ethanol consists of at least two components, initial sensitivity and the development of acute functional tolerance (AFT). Initial sensitivity is measured during the onset of intoxication. In the nematode, *C. elegans*, we observe initial sensitivity at 10 minutes of ethanol exposure. AFT reflects a homeostatic mechanism of the nervous system to rapidly adapt to the intoxicating effects of ethanol that is not a result of ethanol metabolism. In mammals, AFT is observed during a single drug exposure and is measured as a greater behavioral impairment at the beginning of a drinking session than the impairment observed at a similar blood ethanol concentration during the falling phase of blood alcohol concentration. In *C. elegans*, we observe AFT during a single ethanol exposure, in which the animals recover their ability to move despite no decrease in internal ethanol concentration [4].

Several proteins and pathways have been described that modify the development of acute functional tolerance in different species. GABA_A_ receptors isolated from ethanol-treated mice demonstrate acute tolerance *in vitro* [5], and this requires functional Protein Kinase Cε. In addition, Protein Kinase Cε knock out mice show defects in AFT [6]. In *C. elegans*, we have previously shown that the Neuropeptide Y Receptor-like protein, NPR-1, acts to negatively regulate the development of acute functional tolerance. This observation extends to the mouse, where animals with loss of function of various components of the brain NPY pathway recover more rapidly from hypnotic doses of ethanol [7], [8], a measure of the development of AFT in mice [9].

Much work has focused on the effects of ethanol on protein targets, and several excellent candidates for ethanol effector molecules have been identified [10], [11]. As the dominant theory for many years, the lipid fluidity hypothesis of ethanol intoxication proposed an indirect effect of ethanol on membrane proteins via perturbation of membrane lipid structure [12], [13]. While this theory has fallen out of favor for several reasons, including the observation of direct actions of ethanol on proteins, the relevance of lipid-protein interactions in modulating protein function and in modulating direct ethanol-protein effects are increasingly a target of study [14]–[17].

This study is designed to identify the molecular mechanisms for the development of AFT. Here, we report that an unbiased genetic screen for modulators of the development of AFT yielded genes involved in determining levels of triacylglycerols in the animal. Our data support a conclusion that the lipid environment is important in the ability of the animal to modify cell function in the development of AFT to ethanol.

## Results

### Isolation of Mutants Defective in Acute Functional Tolerance

In order to identify the molecular mechanisms underlying acute functional tolerance (AFT) to ethanol, we performed an unbiased genetic screen for animals that are impaired in the ability to develop AFT. We have previously shown that NPR-1 activity antagonizes the development of AFT; in an *npr-1* mutant background, AFT develops more quickly than in wild type, suggesting that NPR-1 function acts to slow the development of AFT [4]. We took advantage of this phenotype of fast and obvious development of AFT in the *npr-1* mutant background to sensitize our screen to make it easy to identify animals with diminished AFT. The advantage of using this sensitized background is that we should be able to identify mutations in which AFT is diminished but not completely abolished, which would be difficult in the N2 wild-type background.

We performed EMS mutagenesis on *npr-1(ky13)* animals, and subjected their F_2_ progeny to a behavioral screen. We treated the animals with ethanol, allowed them time to develop AFT, and then identified animals that had not developed tolerance using a locomotion assay in which animals that could not develop tolerance would be too impaired by ethanol to crawl to an attractive odorant. We isolated animals that had not developed tolerance, allowed them to produce self-progeny, and retested their progeny in a quantitative assay of basal activity and ethanol responses that measured both initial sensitivity and the development of AFT. In this secondary assay, we placed worms on plates that were contained 0 mM or 400 mM of ethanol. Movies were recorded of locomotion at 10 and 30 minutes of treatment, and speed was determined by computer analysis of the movies. We define the decrease in speed at 10 minutes of treatment, relative to untreated animals of the same genotype, as the initial sensitivity of animals to ethanol. We have previously shown that wild-type worms move faster at 30 minutes relative to their speed at 10 minutes of ethanol exposure, and we consider the amount of recovery of speed between 10 and 30 minutes to be the degree of acute functional tolerance. We judged animals to be defective in AFT if they had a reduced recovery of speed at 30 minutes versus the genetic background strain (*npr-1(ky13)* or N2).

**Figure 1:**
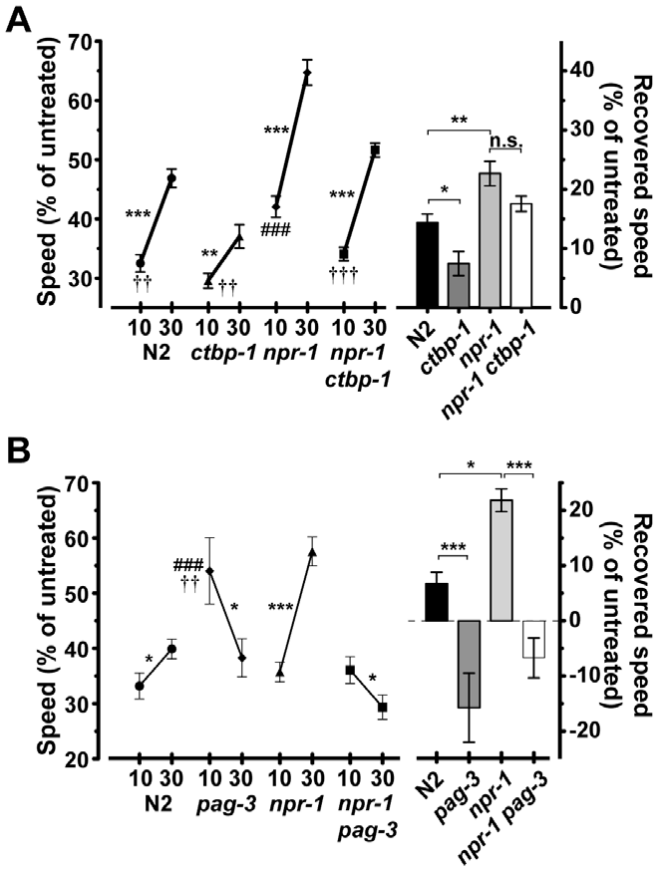
The transcriptional regulators CTBP-1 and PAG-3 are required for the development of AFT. Animals were treated with 0 mM and 400 mM exogenous ethanol. Relative speeds were calculated as treated over untreated speeds. Animals shown in the same graph were tested simultaneously on the same plates. a) Wild-type N2 animals develop AFT, and *npr-1(ky13)* animals are significantly faster developers of AFT. Compared with *npr-1(ky13)*, *npr-1(ky13) ctbp-1(eg613)* animals have decreased initial sensitivity to ethanol; the decrease in development of AFT was not significantly different from *npr-1(ky13)*. *ctbp-1(eg613)* animals in an *npr-1*(+) (N2) background develop significantly less AFT than N2 (*n* = 22). b) PAG-3 is required for the development of AFT. *pag-3(n3098)* animals do not develop AFT, and when tested in the sensitized background, *pag-3(n3098) npr-1(ky13)* animals have decreased initial sensitivity and are slow developers of AFT compared with *npr-1(ky13)* (*n* = 13). Note that the large decrease in speed in *pag-3(n3098)* animals at 30 minutes of ethanol exposure is largely driven by an increase in their untreated speed at 30 minutes, and may not reflect an actual increase in sensitivity to ethanol during that interval. We have not observed this kind of increase in untreated speed in other strains of animals tested, including in the *npr-1(ky13) pag-3(n3098)* double mutant. *pag-3(n3098)* animals do not increase their speed on ethanol over the course of 30 minutes, indicating that they are defective in AFT (at 400 mM: 10 minutes  =  32.4 ± 2.6 µm/sec, 30 minutes  =  33.7 ± 1.3 µm/sec; not significantly different t40  =  0.42, P = 0.67). Error bars are s.e.m. For indicated comparisons: n.s., not significant; *, *P* < 0.05; **, *P* < 0.01; ***, *P* < 0.001; for comparison to N2 at 10 minutes: ###, *P* < 0.001; for comparison to *npr-1(ky13)* at 10 minutes: †, *P* < 0.05; ††, *P* < 0.01; †††, *P* < 0.001.

We tested approximately 2600 mutagenized haploid genomes, and recovered 16 recessive mutations that blunted the development of AFT in the *npr-1(ky13)* mutant background, these identify 14 complementation groups. We pursued molecular characterization of the mutation *eg613* first because of its robust phenotype (Figure 1a).

### Identification of *ctbp-1* as a Mediator of AFT

The *eg613* mutation was mapped to the left arm of the X chromosome using SNP mapping methods with the polymorphic wild strain, CB4856 [18], [19]. Using transformation rescue experiments we identified the gene affected by mutation in the *eg613* mutant strain as *ctbp-1*. We found that the *eg613* mutation is a splice site mutation in the last nucleotide of intron 9 in the open reading frame of the gene *ctbp-1* that is predicted to generate a premature stop codon that results in a truncated protein. A deletion allele of *ctbp-1, ok498*, failed to complement *eg613* for the AFT phenotype, and homozygous *ctbp-1(ok498)* animals phenocopied *eg613* mutants by suppressing the fast development of AFT of *npr-1(ky13)*, tested in a visual assay of speed on ethanol, indicating that *ctbp-1* is disrupted by the *eg613* mutation. The CTBP-1 protein contains a DNA-binding THAP domain, and CTBP-1 has been shown to act as a negative regulator of transcription [20].

In order to determine if *ctbp-1(eg613)* is dependent on a loss of *npr-1* function to modify AFT, we crossed the mutation away from the *npr-1(ky13)* mutation and tested its ability to develop acute tolerance in the presence of wild-type *npr-1*. When we examined the development of AFT in animals carrying the *ctbp-1(eg613)* mutation alone, we found that they had reduced AFT compared with N2 animals (Figure 1a), demonstrating that *ctbp-1* is a component that is generally required for the development of AFT, independent of the genetic background.

**Figure 2:**
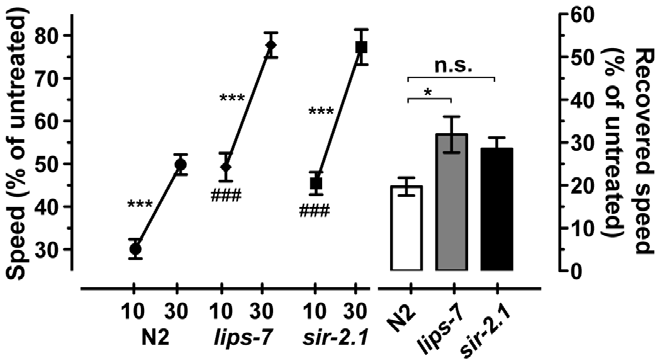
The *ctbp-1*-regulated gene *lips-7* modulates the rate of the development of AFT. Animals were tested on 0 mM and 400 mM exogenous ethanol. Relative speeds were calculated as treated over untreated speeds. All animals were tested simultaneously on the same plates. a) *lips-7(ok3110)* animals are resistant to ethanol at 10 minutes of exposure relative to wild-type N2, and they develop significantly more AFT than wild-type N2 animals (*n* = 13). b) The transcription factor *sir-2.1* is a negative regulator that acts genetically upstream of *ctbp-1* [21]; in a *sir-2.1* mutant strain, loss of negative regulation of *ctbp-1* should result in fast development of AFT. Compared with N2, *sir-2.1(ok434)* mutant animals were resistant to ethanol at 10 minutes of exposure but their apparent increase in development of AFT did not reach statistical significance (*n* = 13). Error bars are s.e.m. For indicated comparisons: n.s., not significant; *, *P* < 0.05; ***, *P* < 0.001; for comparison to N2 at 10 minutes: ###, *P* < 0.001.

The transcriptional regulator *sir-2.1* acts genetically upstream to repress function of *ctbp-1* [21]; we predicted that in a *sir-2.1* mutant strain, loss of negative regulation of *ctbp-1* might result in fast development of AFT. We found that *sir-2.1* mutant animals were resistant to ethanol relative to N2, and, while there was a trend toward fast development of AFT, there was not a statistical difference in rate of AFT from N2 (Figure 2). Together, these results suggest that *ctbp-1* is a regulator of AFT.

### *pag-3* is Required for AFT and *pag-3* was Isolated in the Screen

The transcription factor CTBP-1 has been shown to bind to and act with the zinc-finger transcription factors PAG-3 and ZAG-1 to repress transcription of target genes [20]. We reasoned that if *ctbp-1* mutant animals are defective in AFT because of loss of transcriptional regulation of some effector genes that are cooperatively regulated by these co-repressors, then animals defective in either of the other two co-repressors should also be defective in AFT. Animals mutant in either *pag-3* or *zag-1* have locomotion defects; we were unable to test *zag-1* mutants in our behavioral assays because they are too uncoordinated to allow us to distinguish between locomotion defects induced by ethanol and their baseline uncoordination. The more extreme phenotype of the *zag-1* and *pag-3* mutants compared with *ctbp-1* mutants suggests that those genes have functions in addition to their cooperative role with *ctbp-1*. While *pag-3* mutant animals are also uncoordinated, their locomotion defect is less severe, and we were able to assess them for the development of AFT. We found that the loss-of-function allele *pag-3(n3098)* did not develop AFT, and it also suppressed the fast development of AFT of *npr-1(ky13)* (Figure 1b), suggesting that *ctbp-1(eg613)* is defective in AFT because it fails to regulate transcription, and that this function of *ctbp-1* requires *pag-3*. Since a mutation in *pag-3* was able to suppress the *npr-1*-mediated fast development of AFT, we reasoned that our genetic screen may have identified an allele of *pag-3*. Another mutation isolated in our screen, *bet16*, mapped to the genetic interval containing *pag-3* and failed to complement four alleles of *pag-3* for the AFT phenotype, as tested in visual assays of speed on ethanol. These results indicate that this behavioral screen identified multiple members of a molecular pathway that is involved in the development of AFT.

### Dysregulation of *ida-1′*s Control of Neurosecretion is not Responsible for the AFT Phenotypes of *ctbp-1*

We were interested in determining which genes that are regulated by *ctbp-1* and *pag-3* are responsible for the defect in AFT that we observed in those animals. To date, in *C. elegans*, the only specific regulatory target of *pag-3* that has been reported is the gene *ida-1*, which encodes a protein involved in dense core vesicle release. The loss of negative regulation of *ida-1* in *pag-3* mutants causes increased neurosecretion [22]. We would expect any gene that is important for the shared AFT phenotype in *pag-3* and *ctbp-1* to also be misregulated in the *ctbp-1* mutant. Chen *et al*. [21] performed microarray analysis of a *ctbp-1* mutant, and reported genes with expression differences of 2-fold or more from wild type, and while expression of *ida-1* was not reported to be altered in this study, it remained possible that a more subtle transcriptional dysregulation, which did not meet the criteria for inclusion in that report, may be functionally relevant in the development of AFT. Therefore, we tested whether altering *ida-1* function could be an explanation for the altered rate of development of AFT in *ctbp-1* and *pag-3.* We found that the response of the strong loss-of-function *ida-1(ok409)* mutant animals to ethanol was indistinguishable from that of N2 (Table 1), suggesting that a change in the rate of neurosecretion is not the explanation for the AFT phenotype of animals carrying mutations in these transcriptional regulators. As noted above, we have further observed that *pag-3* mutant animals are mildly uncoordinated, while *ctbp-1* mutants are not, suggesting that this transcriptional repressor may regulate expression of a larger group of genes than those that overlap with *ctbp*-*1*, and *ida-1* may be in that class of differentially regulated genes.

**Table 1:** Mutants tested for initial sensitivity and development of acute functional tolerance to ethanol.

GENE	PROTEIN	PHENOTYPE ofLOSS-of-FUNCTION	INITIAL SENSITIVITY^a^	DEVELOPMENT OF AFT^b^
***ida-1(ok409)*** [22]	Protein tyrosinephosphatase-like receptor	Increase inneurosecretion^c^	Not different from N2	Not different from N2
			N2 (10′) : 28.1 ± 2.4%	N2 (30′): 36.4 ± 2.3%
			*ok409* (10′): 32.1 ± 2.1%	recovery (30′-10′): 8.3 ± 2.3%
			t_6_ = 1.66, *P* = 0.15	*ok409* (30′): 41.8 ± 1.9%
				recovery (30′-10′): 9.7 ± 1.9%
				t_6_ = 0.49, *P* = 0.64
***nhr-49(ok2165)*** ** and** ***nhr-49(gk405)*** [40]	Nuclear hormonereceptor	Increase in fat storage^d^	Not different from N2	No development of AFT
			N2 (10′): 30.3 ± 1.4%	N2 (30′): 34.8 ± 3.3%
			*ok2165* (10′): 30.3 ± 3.0%	recovery (30′-10′): 4.5 ± 3.7%
			t_7_ = 0.002, *P* = 0.99	*ok2165* (30′): 28.0 ± 3.2%
			N2 (10′): 34.9 ± 2.6%	recovery (30′-10′): -2.3 ± 1.4%
			*gk405* (10′): 38.8 ± 3.7%	N2 (30′): 47 ± 4.0%
			t_6_ = 0.97, *P* = 0.37	recovery (30′-10′): 11.7 ± 2.5%
				*gk405* (30′): 38.6 ± 3.8%
				recovery (30′-10′): -0.2 ± 3.1%
***bbs-1(ok1111)*** [41]	Ortholog to human BBS1	Increase in fat storage^d^	More sensitive than N2	Faster development of AFT relative to N2
			N2 (10′): 31.4 ± 3.4%	N2 (30′): 43.1 ± 2.1%
			*ok1111* (10′): 15.2 ± 0.95%	recovery (30′-10′): 11.7 ± 2.5%
			t_8_ = 4.5, *P* <0.01	*ok1111* (30′): 32.0 ± 2.1%
				recovery (30′-10′): 16.7 ± 2.0%
				t_8_ = 2.9, *P* < 0.05
***tub-1(ok1972)*** [42]	tubby	Increase in fat storage^d^	Not different from N2	Not different from N2
			N2 (10′): 34.9 ± 3.5%	N2 (30′): 48.9 ± 2.9%
			*ok1972* (10′): 28.3 ± 2.5%	recovery (30′-10′): 13.8 ± 1.9%
			t_5_ = 1.4, *P* = 0.21	*ok1972* (30′): 46.6 ± 4.9%
				recovery (30′-10′): 15.1 ± 2.0%
				t_5_ = 0.47, *P* = 0.65
***fat-7(wa36); fat-5(tm420)*** [43]	Fatty acid desaturases	Decrease in fat storage^d^	Not different from N2	Faster development of AFT relative to N2
			N2 (10′): 32.2 ± 1.6%	N2 (30′): 35.3 ± 3.2%
			*wa36;tm420* (10′): 30.3 ± 2.8%	recovery (30′-10′): 6.5 ± 1.6%
			t_11_ = 0.80, *P* = 0.44	*wa36;tm420* (30′): 44.6 ± 2.5%
				recovery (30′-10′): 14.2 ± 1.7%
				t_11_ = 6.7, *P* < 0.0001
***sbp-1(ep79)*** [44]	Homolog to mammalianSREBP	Decrease in fat storage^d^	Less sensitive than N2^ e^	No development of AFT
			N2 (10′): 32.4 ± 2.6%	N2 (30′): 43.6 ± 2.8%
			*ep79* (10′): 45.9 ± 4.2%	recovery (30′-10′): 11.2 ± 2.4%
			t_6_ = 3.0, *P* < 0.05	*ep79* (30′): 37.9 ± 5.5%
				recovery (30′-10′): -8.0 ± 8.5%

a The degree of initial sensitivity was determined as the percent of untreated speed after 10 minutes of ethanol exposure. Significance was determined relative to the effect of ethanol on wild-type N2 animals treated on the same plates using paired two-tailed t-tests. ^b^The development of acute functional tolerance (AFT) was determined by comparing the rate of locomotion at 30 minutes vs. 10 minutes of ethanol exposure. If the mutant strain developed significant tolerance, we compared its rate of development of tolerance to the rate of wild-type N2 animals treated on the same plates using unpaired two-tailed t-tests. ^c^ *ida-1* is known to be negatively regulated by *pag-3*; the response of the strong loss of function *ida-1(ok409)* mutant animals to ethanol was indistinguishable from that of N2, suggesting that a change in neurosecretion is not the explanation for the AFT phenotype of *ctbp-1* and *pag-3*. ^d^Genetic manipulation of fat stores by mutations in the genes *nhr-49, bbs-1, tub-1, fat-7* and *fat-5,* and *sbp-1* did not alter ethanol sensitivity or AFT in a consistent manner, suggesting that fat levels themselves do not influence the rate of development of AFT. ^e^ *sbp-1* mutant animals have a basal speed that is significantly slower than N2 (Table S1). For this reason, the magnitude and interpretation of the ethanol responses of this strain may be subject to a floor effect.

### *ctbp-1* Regulates AFT through Repression of *lips-7* Expression

CTBP-1 has been demonstrated to regulate the transcription of approximately 200 genes [21]. Chen *et al* [21] reported that *ctpb-1(ok498)* mutants have an extended lifespan, and that the loss of regulation of the *lips-7* triacylglycerol (TAG) lipase in the *ctbp-1* mutant could explain this aspect of the *ctbp-1* mutant phenotype. In *ctbp-1(ok498)* mutant animals, *lips-7* message is increased, and the resulting lipase overexpression caused animals to accumulate 16.8% less TAGs, and animals in which *lips-7* had been inactivated by RNA interference had dramatically increased levels of TAGs [21]. We hypothesized that if the effect of loss of function of *ctbp-1* on ethanol response, like its effect on lifespan, is due to its failure to down-regulate *lips-7*, then loss of *lips-7* should have the opposite phenotype to loss of *ctbp-1*; that is, *lips-7* mutants should display ethanol resistance and/or fast development of AFT. We found that *lips-7(ok3110)* mutant animals are both resistant to ethanol and fast developers of AFT, indicating that regulation of TAG levels is important for the development of AFT (Figure 2a). These results support the notion that the effect on lipase regulation *via lips-7* is a mechanism of *ctbp-1′*s effect on AFT (Figure 2b).

### Fat Stores do not Regulate AFT

Regulation of the lipase gene *lips-7* has a significant effect on the development of AFT. In one model of how this lipase function affects AFT, fat levels in the animals could regulate ethanol effects because TAGs are the main stored fat in *C. elegans.* To test this hypothesis, we altered fat levels in the animals using genetic manipulations, and tested the effect on ethanol sensitivity and AFT development in our locomotion assay. If the changes in the level of stored fat in *ctbp-1* and *lips-7* mutants is the cause of the defect in AFT, then decreasing fat levels through genetic manipulation should cause a decrease in AFT (mimicking *ctbp-1(eg613)* mutants), while increasing fat levels should cause animals to be resistant to ethanol or fast developers of AFT (similar to *lips-7* mutant animals). We tested several genes involved in regulating the levels of stored fat; mutations in these genes either yield increases or decreases in stored fat (Table 1). While several changes in fat levels did not cause an AFT phenotype, for those that did alter AFT, there was no consistent correlation between fat levels and the ability to develop AFT (Table 1). These data suggest that, by themselves, levels of stored fat do not predict the ability of the animal to develop AFT.

### The Development of AFT is Cholesterol Dependent

**Figure 3:**
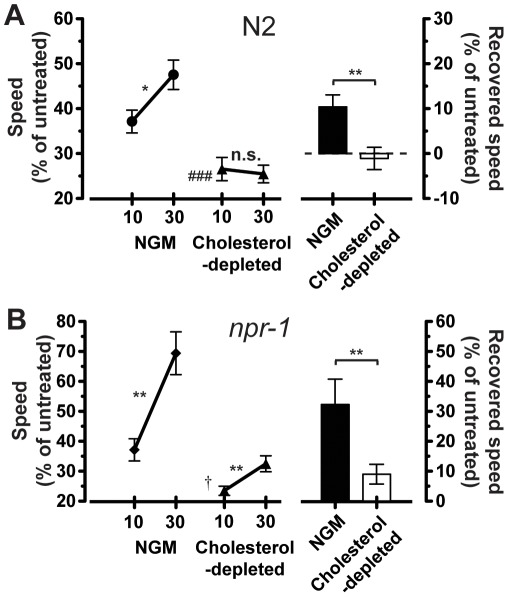
Disruption of the plasma membrane through cholesterol starvation eliminates the ability to develop AFT. Worms require exogenous cholesterol for plasma membrane mediated processes such as clathrin-independent endocytosis. Young adult animals that were starved of exogenous cholesterol during their development were tested on 0 mM and 400 mM ethanol. Relative speeds were calculated as treated over untreated. Animals shown in the same graph were tested simultaneously on the same plates. a) Wild-type N2 animals develop AFT when reared on cholesterol-containing (NGM) plates, but do not develop AFT when starved of cholesterol (*n* = 14). b) *npr-1(ky13)* animals develop AFT when reared on NGM plates, but develop reduced levels of AFT when starved of cholesterol (*n* = 7). Together, these results suggest that the development of AFT requires cholesterol in the membrane. Error bars are s.e.m. For indicated comparisons: n.s., not significant; *, *P* < 0.05; **, *P* < 0.01; for comparison to N2 on NGM at 10 minutes: ###, *P* < 0.001; for comparison to *npr-1(ky13)* on NGM at 10 minutes: †, *P* < 0.05.

In addition to their role in fat storage, TAGs are also components of the lipid membrane. To analyze the involvement of membrane environment in the development of AFT, we manipulated the plasma membrane by depleting the worms of cholesterol, a component of membrane lipid rafts [23]. *C. elegans* are cholesterol auxotrophs; all of their cholesterol is captured through their diet, and acute effects of cholesterol depletion include a suppression of clathrin-independent endocytosis in neurons, a lipid raft-mediated process [24], suggesting that cholesterol is involved in the development or maintenance of particular membrane structures. If AFT requires specific membrane microdomain structures that include cholesterol, then altering the amount of cholesterol available to the animals may modify their ability to develop tolerance. We found that N2 and *npr-1(ky13)* animals reared on cholesterol-depleted media demonstrated a substantial suppression of the development of AFT (Figure 3a and 3b). In contrast, adding additional cholesterol to the medium did not affect the ability of animals to develop AFT (not shown). Together, these data suggest that the structure of the lipid bilayer is essential for the development of tolerance.

### The Development of AFT Requires SLO-1

**Figure 4:**
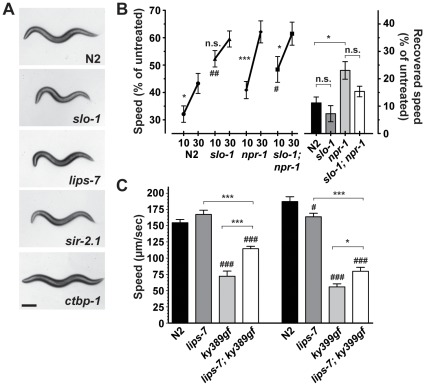
SLO-1 protein function is modulated by *lips-7* function. a) Representative micrographs of crawling *C. elegans*, when the animal’s head is at the dorsal most point in the propagation of its waveform. Anterior is left, scale bar is 200 µm. Wild-type N2 demonstrates the normal sinusoidal body posture of a crawling worm. *slo-1(eg142)*, *lips-7(ok3110)*, and *sir-2.1(ok434)* demonstrate a similar exaggerated, loopy, body posture. In contrast, *ctbp-1(eg613)* has a flattened body posture. b) SLO-1 function is required for normal AFT. Worms were treated with 400 mM exogenous ethanol. *slo-1(eg142)* null mutant animals do not develop significant AFT, whereas *slo-1(eg142); npr-1(ky13)* double mutants do develop tolerance. The degree of recovered speed of *slo-1(eg142); npr-1(ky13)* did not achieve statistical significance compared with *npr-1(ky13)*, although there was a strong trend for an attenuated response compared with the robust development of AFT by *npr-1(ky13)* (*n* = 14). These data suggest that SLO-1 is not the sole mediator of the development of AFT in the worm. c) Loss of *lips-7* can modulate function of *slo-1* gain-of-function alleles. *slo-1(ky398gf)* and *slo-1(ky399gf)* mutations increase the open probability or open time of the SLO-1 channel, resulting in a slow locomotion phenotype [29]. Loss of *lips-7* significantly suppresses the slow movement phenotype of both *slo-1(gf)* alleles in the absence of ethanol (*n* = 6 for *slo-1(ky389gf)*; *n* = 9 for *slo-1(ky399gf)*) indicating that the increase in TAGs in *lips-7* can cause a change in the function of the membrane channel, SLO-1. These results suggest that *lips-7* alters SLO-1 function through a plasma membrane mechanism. Error bars are s.e.m. For indicated comparisons: n.s., not significant; *, *P* < 0.05; ***, *P* < 0.001; for comparison to N2: #, *P* < 0.05; ##, *P* < 0.01; ###, *P* < 0.001.

Both the manipulation of cholesterol and the lipid constituents of the membrane environment are known to modulate the ethanol effect on the mammalian BK (KCNMA1/Slo1) potassium channel, an ethanol target protein[14], [17], [25]–[28]. In worms, ethanol activates SLO-1 *in vivo*, causing a large efflux of potassium ions, hyperpolarizing the cell and depressing neuronal excitability, which is a major cause of intoxication in *C. elegans* [29]. The mammalian BK channel is also activated by ethanol, and this effect, as well as the channel’s basal activity, depends on the thickness of the lipid bilayer; channels that reside in thicker membrane are less basally active and are less activated by ethanol [17], [28], suggesting that movement of the BK channel into and out of areas of thicker membrane microenvironments may allow for very fast modulation of the effects of ethanol on this channel [28]. We hypothesized that this may be one mechanism by which worms develop AFT to ethanol. If AFT requires modulation of SLO-1 function, then complete loss of SLO-1 should reduce or eliminate the development of tolerance. In an otherwise wild-type background, null *slo-1(eg142)* mutant animals are quite resistant to the sedative effects of ethanol on locomotion, and, importantly, do not develop significant tolerance to ethanol (Figure 4b). We asked if loss of *slo-1* completely eliminated the ability of animals to develop tolerance, even in a genetic background of fast development of tolerance. In the sensitized *npr-1(ky13)* mutant background, *slo-1(eg142)* animals, that lack SLO-1, are able to develop a degree of tolerance (Figure 4b), indicating that modulation of the ethanol effect on SLO-1 occurs during AFT but that SLO-1 is not the sole mediator of AFT.

### *ctbp-1* and *lips-7* Modulate the Function of SLO-1

If modulation of SLO-1 activity represents a significant component of AFT then it is possible that the increased TAG levels associated with loss of *lips-7* or *sir-2.1* function are acting to either protect the SLO-1 protein from ethanol or are reducing overall activity of the SLO-1 protein, or both. If the action of this increase in TAGs is to reduce overall activity of SLO-1 then we predicted that we should be able to observe an inhibitory effect on SLO-1 function in the *lips-7* and *sir-2.1* mutants in the absence of ethanol. Reduced SLO-1 function in the *slo-1* mutant causes an increase in the amplitude of the locomotion waveform (loopy motion phenotype, Figure 4a), and we observed that both *lips-7* and *sir-2.1* mutants are loopy (Figure 4a). In contrast, we predicted that in a *ctbp-1* mutant, in which there is a decrease in TAGs, there should be an increase in SLO-1 activation, which should lead to the opposite phenotype, a flattened body waveform. Consistent with the prediction, we found that *ctbp-1* mutants are flat relative to N2 (Figure 4a).

We were able to test more directly whether or not loss of *lips-7* is able to modulate the function of SLO-1 in the absence of ethanol, using special alleles of *slo-1*. Two gain-of-function alleles of *slo-1* that enhance the open probability and open time of the channel have been described [29]. These animals have a slow locomotion phenotype, which shares characteristics with the phenotype of ethanol intoxication. We hypothesized that *lips-7* might suppress these gain-of-function defects if the increase in levels of TAGs reduces the activity of the channel. We tested the locomotion of *slo-1(ky389gf); lips-7(ok3110)* and *slo-1(ky399gf); lips-7(ok3110)* animals and found that loss of *lips-7* was able to significantly rescue the locomotion defect of both *slo-1* gain-of-function mutations (Figure 4c). These data strongly suggest that changes in TAG levels resulting from increased or decreased LIPS-7 activity are able to influence the activity of a transmembrane protein (SLO-1) both in its basal state and in the presence of ethanol.

## Discussion

We have used a genetic screen for mutations that result in defects in the development of AFT to identify two transcriptional co-repressors, *ctbp-1* and *pag-3*, that regulate the ability of animals to develop AFT. Transcriptional repression of the *lips-7* TAG lipase is required for normal development of AFT, and loss of *lips-7* enhances the rate and degree of AFT, indicating that action of this lipase inhibits the development of AFT. One component of the normal development of AFT appears to involve modulation of the ethanol effect on the transmembrane ethanol target protein SLO-1, and the function of SLO-1 gain-of-function mutations is modulated by the action of *lips-7*, suggesting that there is a role for TAGs in the regulation of SLO-1 function. Depletion of cholesterol impaired the development of AFT and, while cholesterol is used both as a component of the plasma membrane and as a precursor to steroid synthesis in the worm, taken together, these data suggest a role for membrane microdomain structure in AFT.

These results lead us to propose a model for a mechanism for fast adaptations associated with the development of AFT: the effects of ethanol on a membrane protein, such as SLO-1, are countered by moving the protein into or out of specific membrane microdomains to achieve a more normal function of the neuron in the presence of ethanol. In this model, cholesterol and the TAGs on which LIPS-7 acts would constitute components of these microdomains that reduce the ethanol effect on the target protein, either by protecting the protein from ethanol or by changing the protein’s activity in opposition to the ethanol effect. An increase in the levels of these TAGs might lead to an increase in the size or quantity of these protective microdomains, thereby increasing the potential for minimizing ethanol’s effects on target proteins; decreases in the level of these TAGs would have the opposite effect on AFT. This model accounts for the altered rates and development of AFT in mutants for the *ctbp-1, pag-3* and *lips-7* genes, which regulate levels of these TAGs in different directions.

While we favor a model of a direct effect of lipid microdomains in modulation of SLO-1 activity, as is seen for mammalian SLO-1/BK channels (reviewed by Treistman and Martin [30]), the data presented here are also compatible with a model in which lipid modulation of activity of neuronal function occurs independently of SLO-1 function. For example, altering the level of TAGs might increase the excitability of cholinergic motor neurons or decrease the excitability of GABAergic motor neurons, both effects might lead to a decreased ethanol response. Loss of *lips-7* function, which increases TAG levels, results in a loopy locomotion phenotype, acute ethanol resistance and a more rapid development of AFT. These phenotypes are consistent with an increase in the overall excitability of the locomotor cholinergic circuitry. However, we have shown previously with mutants that enhance the activity of the cholinergic neurons, that the degree of neuronal excitability and the degree of ethanol sensitivity are not well correlated [29]. Effects by TAGs in a SLO-1-dependent manner and general neuronal excitability effects are not mutually exclusive models, and in fact these two postulated lipid effects may act in concert to modify ethanol response at the behavioral level.

While our results do not directly address the question of the causative microdomains in the membrane, one obvious candidate is the lipid raft, a cholesterol- and sphingomyelin-rich microdomain in the plasma membrane. We show that cholesterol depletion results in a significant reduction in the development of AFT. Mammalian SLO-1/BK function is known to be modulated by the cholesterol environment in which it resides in the membrane [14], [25]–[27], and the mammalian BK channel is found in lipid rafts [31]. Recent studies suggest that the modulation of channel function by cholesterol is through a specific interaction with a protein surface on the SLO-1/BK channel itself rather than a secondary effect of cholesterol’s effect on lipid packing [25], [27].

There is increasing awareness of the modulation of function of membrane proteins by their interaction with lipids. This model of proteins moving between different lipid microenvironments during AFT might include the effects of ethanol on multiple ethanol responsive proteins in addition to SLO-1. For instance, ethanol treatment inhibits localization of the proteins Lck, ZAP70, LAT and PLCγ1 to lipid rafts in T lymphocytes [15]. Another intriguing candidate for a protein whose function is may be modulated by its location in rafts is H-ras, which is known to be involved in the dynamic response of NMDA receptors to ethanol [32]. In its inactive form, H-ras resides in lipid rafts, and when activated, it must move out of rafts to efficiently activate its target Raf [33].

Importantly, this mechanism of modulation of protein function may reflect a more general process used in maintaining homeostasis in neurons. The mechanisms of development of AFT to ethanol are likely to represent processes that neurons use to modulate their function in response to depressive stimuli in general.

An individual’s level of response to ethanol is dependent on the AFT process. We hypothesize that subtle alterations in plasma membrane structure, perhaps through natural genetic variation in the lipid metabolism machinery, could lead to differences in rates of development of AFT between individuals, which may contribute to predisposition to alcohol dependence (AD) [1]. Additional support for this hypothesis comes from the observation that membranes isolated from the brains of rats selectively bred for high and low ethanol sensitivity showed differences in their ethanol responsive phenotypes as measured by both membrane protein mobility and membrane thickness [34].

Recently, a human genome-wide association study of AD in an Australian population identified a SNP in the human homolog of *ctbp-1*, called CTBP2, as the marker with the most significant *P* value for association with AD [35]. Significantly, the mammalian CTBP2 protein also acts as a transcriptional repressor, and has recently been shown to play a role in regulating genes with roles in lipid metabolism in adipocytes [36]. Variation in the function of this gene may have more general effects on lipid membrane composition and could impact responses to alcohol. We suggest that it may be fruitful to further examine genes involved in cholesterol and lipid biosynthesis in vertebrate models and human genetic association studies of alcohol dependence.

## Materials and Methods

### Worm Husbandry

*C. elegans* strains were maintained as described [37]. Strains used in this study were: N2 (var. Bristol), AX201 *npr-1(ky13)*, BZ613 *ctbp-1(eg613)* (4×outcrossed), RB733 *ctbp-1(ok498)*, BZ846 *ctbp-1(ok498); npr-1(ky13)*, MT8987 *pag-3(n3098)*, JCB40 *pag-3(n3098); npr-1(ky13)*, JCB25 *pag-3(bet16); npr-1(ky13)* (1×outcrossed), VC226 *ida-1(ok409)*, RB2287 *lips-7(ok3110)*, VC199 *sir-2.1(ok434)*, RB1716 *nhr-49(ok2165)*, VC870 *nhr-49(gk405)*, VC837 *bbs-1(ok1111)*, RB1600 *tub-1(ok1972)*, BX110 *fat-7(wa36); fat-5(tm420)*, CE541 *sbp-1(ep79)*, BZ142 *slo-1(eg142)*, JCB95 *slo-1(ky389)* (1×outcrossed), JCB96 *lips-7(ok3110); slo-1(ky389)*, JCB97 *slo-1(ky399)* (1×outcrossed), and JCB98 *lips-7(ok3110)*; *slo-1(ky399)*.

### Genetic Screen

*npr-1(ky13)* animals were mutagenized in 47 mM ethyl methanesulfonate (EMS) for 4 hours. Second generation progeny were screened for failure to develop AFT: Worms were incubated for 90 minutes in 300 mM ethanol in M9, then were placed on one side of a 10 cm Petri plate filled with assay agar [38] and 300 mM ethanol. Worms were enticed to move across the plate to a spot of chemoattractant (1 µL of 1∶200 benzaldehyde:ethanol). In these conditions, all *npr-1(ky13)* animals are able to develop sufficient AFT to be able to move to the spot of chemoattractant. After 90 minutes, worms that had not reached the chemoattractant were picked individually and allowed to generate self-progeny. These progeny were tested in a secondary screen for the development of AFT; animals that failed to develop AFT were kept.

### Analysis of Speed

Speed was analyzed as described previously [4], with minor modifications: Briefly, assay plates were dried for two hours at 37°C with lids off on the day of the assay. Copper rings were melted into the surface of the plates, and two hours before the beginning of the assay, ice-cold 100% ethanol was added to plates to a final concentration in the agar of 0 mM or 400 mM. Age-matched first day adult animals were acclimated to the lack of food in the assay for a period of 30 minutes by moving them to unseeded dried plates. Ten worms of each test strain were moved into a copper ring on an assay plates and movies were recorded of their movement at 10 and 30 minutes of exposure. Only strains that were tested in the same assay on the same plates were compared to each other. Movies were made on an Olympus SZX-7 stereo microscope (magnification of 0.8x with a 0.5x objective to make a large enough field of view) using a Retiga 4000R camera (QImaging) and ImagePro Plus (6.2) (MediaCybernetics) software. 2-minute recordings (1 frame per second) were captured, and the speed of each worm was tracked using ImagePro plus software and an average speed for each group of 10 animals was calculated.

### Assessment of Ethanol Response

We assess ethanol response in three ways:

1. Initial Sensitivity: We compare the degree of depression of speed (relative speed) at 10 minutes to that of the control strain. A statistical difference at this timepoint reflects a change in initial sensitivity.
2. Development of Acute Functional Tolerance: We compare the speed of a strain at 30 minutes versus 10 minutes to determine if the strain had developed AFT. A statistically significant increase in speed at 30 minutes vs. 10 minutes is AFT.
3. Comparison of AFT with the control strain: We compare the amount of speed recovered versus the control strain. Statistically significant differences in the amount of recovered speed reflects a difference in the degree of AFT.

### Statistics

Animals that were compared were tested on the same plates under identical conditions. Untreated and treated raw speeds are shown in Table S1. Relative speeds (treated/untreated X 100) for ethanol effects were calculated and used for all strains in statistical comparisons. Statistics were performed using Prism 5.0 (GraphPad). Comparisons of 10-minute treated speed, comparisons of recovered speed (treated speed at 30 minutes minus treated speed at 10 minutes), and comparison of untreated speed on NGM versus cholesterol-depleted media were made using 1-way ANOVA, with Tukey’s multiple comparison posthoc tests. Development of AFT was tested by unpaired two-tailed t-test.

### Cholesterol Starvation

Cholesterol-depleted media was prepared as described previously [39].

## Supporting Information

Table S1 **Comparison of untreated speeds and treated speeds (400 mM ethanol) for strains in each experiment used to calculate relative treated speeds.** All strains were compared with N2, except for Figure 1b and Figure 3b, where *npr-1(ky13)* is the comparison strain. The data was analyzed using 2-way repeated measures ANOVA. If the ANOVA was significant for the strain comparison, Bonferroni multiple comparison post-hoc tests were used to determine the significance of differences between the test strain at 10 minutes and at 30 minutes of ethanol exposure compared with the comparison strain.
(DOC)
