# Supplementary material for: Lipid Environment Modulates the Development of Acute Tolerance to Ethanol in Caenorhabditis elegans
Source: PLoS One. 2012 May 4;7(5):e35192. doi: 10.1371/journal.pone.0035192 (PMC3344825; doi:10.1371/journal.pone.0035192)
Supplement: Table S1 — Comparison of untreated speeds and treated speeds (400 mM ethanol) for strains in each experiment used to calculate relative treated speeds. All strains were compared with N2, except for Figure 1b and Figure 3b, where npr-1(ky13) is the comparison strain. The data was analyzed using 2-way repeated measures ANOVA. If the ANOVA was significant for the strain comparison, Bonferroni multiple comparison post-hoc tests were used to determine the significance of differences between the test strain at 10 minutes and at 30 minutes of ethanol exposure compared with the comparison strain. (DOC) [file pone.0035192.s001.doc]

**Table S1**

**Comparisons of untreated speeds for strains used in each experiment and treated speeds (400 mM) for those strains used to calculate relative treated speeds.**

| **Figure/Table** | **Strain/Treatment** | **Time (min)** | **Mean untreated**  **speed ± s.e.m.**  **(µm/sec) (*n*)** | **Significantly different from N2 or comparison strain** | **Mean treated**  **speed ± s.e.m.**  **(µm/sec) (*n*)** |
| --- | --- | --- | --- | --- | --- |
| **Figure 1a** | N2 | 10 | 199.6 ± 5.9 (22) |  | 64.2 ± 2.5 (22) |
|  |  | 30 | 202.7 ± 4.3 (22) |  | 93.9 ± 2.7 (22) |
|  | *ctbp-1(eg613)* | 10 | 115.7 ± 3.5 (22) | *P* < 0.001 | 34.8 ± 1.8 (22) |
|  |  | 30 | 118.1 ± 4.6 (22) | *P* < 0.001 | 43.0 ± 1.7 (22) |
|  | *npr-1(ky13)* | 10 | 222.1 ± 3.9 (22) | *P* < 0.01 | 93.9 ± 3.8 (22) |
|  |  | 30 | 215.7 ± 4.7 (22) | n.s. | 137.4 ± 4.9 (22) |
|  | *npr-1(ky13) ctbp-1(eg613)* | 10 | 171.7 ± 2.9 (22) | *P* < 0.001 | 58.5 ± 1.6 (22) |
|  |  | 30 | 163.1 ± 2.9 (22) | *P* < 0.001 | 84.1 ± 1.6 (22) |
| **Figure 1b** | N2 | 10 | 183.6 ± 5.6 (24) |  | 57.9 ± 2.9 (24) |
|  |  | 30 | 196.3 ± 2.9 (24) |  | 77.9 ± 3.5 (24) |
|  | *npr-1(ky13)* | 10 | 227.9 ± 5.3 (24) | *P* < 0.001 | 80.2 ± 3.4 (24) |
|  |  | 30 | 212.0 ± 4.5 (24) | n.s. | 121.2 ± 5.1 (24) |
|  | *pag-3(n3098)* | 10 | 68.0 ± 4.3 (24) | *P* < 0.001 | 32.4 ± 2.6 (24) |
|  |  | 30 | 96.2 ± 4.8 (24) | *P* < 0.001 | 33.7 ± 1.3 (24) |
|  | *npr-1(ky13) pag-3(n3098)* | 10 | 106.3 ± 6.1 (13) | *P* < 0.001 | 29.1 ± 1.9 (24) |
|  |  | 30 | 115.5 ± 6.8 (13) | *P* < 0.001 | 27.6 ± 1.3 (24) |
| **Figure 2** | N2 | 10 | 189.8 ± 9.0 (11) |  | 56.1 ± 3.4 (11) |
|  |  | 30 | 182.1 ± 6.9 (11) |  | 89.6 ± 2.6 (11) |
|  | *lips-7(ok3110)* | 10 | 208.5 ± 3.8 (11) | n.s. | 98.3 ± 6.2 (11) |
|  |  | 30 | 185.5 ± 5.2 (11) | n.s. | 143.4 ± 7.2 (11) |
|  | *sir-2.1(ok434)* | 10 | 183.7 ± 4.5 (11) | n.s. | 86.5 ± 4.5 (11) |
|  |  | 30 | 169.1 ± 8.0 (11) | n.s. | 129.3 ± 5.0 (11) |
| **Figure 3a** | N2 NGM | 10 | 166.9 ± 7.0 (11) |  | 37.4 ± 2.6 (11) |
|  |  | 30 | 175.8 ± 6.8 (11) |  | 51.7 ± 2.6 (11) |
|  | N2 cholesterol-depleted | 10 | 121.5 ± 6.3 (11) | *P* < 0.001 | 25.8 ± 2.7 (11) |
|  |  | 30 | 122.2 ± 3.7 (11) | *P* < 0.001 | 25.0 ± 1.9 (11) |
| **Figure 3b** | *npr-1(ky13)* NGM | 10 | 207.7 ± 4.8 (9) |  | 47.1 ± 7.5 (9) |
|  |  | 30 | 208.5 ± 13.0 (9) |  | 88.6 ± 13.3 (9) |
|  | *npr-1(ky13)* | 10 | 156.1 ± 4.7 (9) | *P* < 0.001 | 26.6 ± 2.4 (9) |
|  | cholesterol-depleted | 30 | 142.7 ± 6.1 (9) | *P* < 0.001 | 35.6 ± 2.8 (9) |
| **Figure 4b** | N2 | 10 | 216.9 ± 9.9 (14) |  | 69.6 ± 7.5 (14) |
|  |  | 30 | 207.1 ± 11.5 (14) |  | 88.2 ± 8.6 (14) |
|  | *slo-1(eg142)* | 10 | 142.9 ± 4.6 (14) | *P* < 0.001 | 74.2 ± 4.2 (14) |
|  |  | 30 | 153.6 ± 5.4 (14) | *P* < 0.001 | 91.5 ± 5.5 (14) |
|  | *npr-1(ky13)* | 10 | 247.6 ± 6.6 (14) | n.s. | 100.2 ± 7.1 (14) |
|  |  | 30 | 232.4 ± 11.9 (14) | n.s. | 141.4 ± 9.3 (14) |
|  | *slo-1(eg142);npr-1(ky13)* | 10 | 197.7 ± 7.3 (14) | n.s. | 93.3 ± 7.3 (14) |
|  |  | 30 | 180.1 ± 10.8 (14) | n.s. | 106.4 ± 5.7 (14) |
| **Table 1** | N2 | 10 | 196.9 ± 3.9 (7) |  | 55.6 ± 5.5 (7) |
|  |  | 30 | 193.6 ± 4.9 (7) |  | 70.6 ± 5.0 (7) |
|  | *ida-1(ok409)* | 10 | 151.2 ± 5.7 (7) | *P* < 0.001 | 48.1 ± 2.7 (7) |
|  |  | 30 | 147.9 ± 4.1 (7) | *P* < 0.001 | 61.8 ± 3.3 (7) |
|  | N2 | 10 | 196.5 ± 20.1 (8) |  | 58.9 ± 6.0 (8) |
|  |  | 30 | 185. 2 ± 14.9 (8) |  | 67.5 ± 10.9 (8) |
|  | *nhr-49(ok2165)* | 10 | 148.9 ± 3.0 (8) | *P* < 0.05 | 45.0 ± 4.5 (8) |
|  |  | 30 | 141.5 ± 4.6 (8) | *P* < 0.05 | 39.5 ± 4.6 (8) |
|  | N2 | 10 | 176.2 ± 10.7 (7) |  | 60.8 ± 4.1 (7) |
|  |  | 30 | 183.0 ± 13.1 (7) |  | 82.4 ± 3.8 (7) |
|  | *nhr-49(gk405)* | 10 | 118.3 ± 8.3 (7) | *P* < 0.01 | 44.9 ± 3.3 (7) |
|  |  | 30 | 117.0 ± 8.3 (7) | *P* < 0.001 | 43.7 ± 2.5 (7) |
|  | N2 | 10 | 179.4 ± 10.4 (9) |  | 54.2 ± 4.0 (9) |
|  |  | 30 | 190.7 ± 8.7 (9) |  | 81.7 ± 4.8 (9) |
|  | *bbs-1(ok1111)* | 10 | 174.7 ± 8.0 (9) | n.s. | 26.3 ± 1.7 (9) |
|  |  | 30 | 174.9 ± 5.9 (9) | n.s. | 56.7 ± 5.1 (9) |
|  | N2 | 10 | 184.6 ± 7.0 (6) |  | 64.3 ± 6.5 (6) |
|  |  | 30 | 187.2 ± 5.0 (6) |  | 91.3 ± 4.7 (6) |
|  | *tub-1(ok1972)* | 10 | 134.5 ± 8.0 (6) | *P* < 0.001 | 37.8 ± 3.4 (6) |
|  |  | 30 | 137.4 ± 7.3 (6) | *P* < 0.001 | 63.1 ± 5.6 (6) |
|  | N2 | 10 | 202.6 ± 8.6 (12) |  | 65.4 ± 4.6 (12) |
|  |  | 30 | 203.2 ± 8.4 (12) |  | 80.9 ± 5.7 (12) |
|  | *fat-7(wa36);fat-5(tm420)* | 10 | 229.9 ± 5.8 (12) | *P* < 0.05 | 69.4 ± 6.5 (12) |
|  |  | 30 | 215.8 ± 4.7 (12) | n.s. | 95.9 ± 5.6 (12) |
|  | N2 | 10 | 227.5 ± 9.1 (7) |  | 74.1 ± 6.9 (7) |
|  |  | 30 | 216.9 ± 8.5 (7) |  | 95.2 ± 7.8 (7) |
|  | *sbp-1(ep79)* | 10 | 63.5 ± 5.4 (7) | *P* < 0.001 | 28.8 ± 3.2 (7) |
|  |  | 30 | 72.0 ± 4.7 (7) | *P* < 0.001 | 26.3 ± 2.6 (7) |

All strains were compared with N2, except for Figure 1b and Figure 3b, where *npr-1(ky13)* is the comparison strain. The data was analyzed using 2-way repeated measures ANOVA. If the ANOVA was significant for the strain comparison, Bonferroni multiple comparison post-hoc tests were used to determine the significance of differences between the test strain at 10 minutes and at 30 minutes compared with the comparison strain.
